# Scenarios of maternal mortality reduction by 2030 in the Americas: insights from its tempo

**DOI:** 10.1186/s12939-023-01938-y

**Published:** 2023-06-28

**Authors:** Antonio Sanhueza, Oscar J. Mujica, Patricia N. Soliz, Adrienne L. Cox, Bremen de Mucio

**Affiliations:** 1 Department of Evidence and Intelligence for Action in Health, Pan American Health Organization, PAHO/WHO, Washington, DC USA; 2 Latin American Center for Perinatology, Women’s Health, and Reproductive Health (CLAP/WR), Pan American Health Organization, PAHO/WHO, Montevideo, Uruguay

**Keywords:** Maternal mortality, Health equity, Trends, Sustainable Development Goals, Americas

## Abstract

**Background:**

The enduring threat of maternal mortality to health worldwide and in the Americas has been recognized in the global and regional agendas and their targets to 2030. To inform the direction and amount of effort needed to meet those targets, a set of equity-sensitive regional scenarios of maternal mortality ratio (MMR) reduction based on its *tempo* or speed of change from baseline year 2015 was developed.

**Methods:**

Regional scenarios by 2030 were defined according to: i) the MMR average annual rate of reduction (AARR) needed to meet the global (70 per 100,000) or regional (30 per 100,000) targets and, ii) the horizontal (proportional) or vertical (progressive) equity criterion applied to the cross-country AARR distribution (i.e., same speed to all countries or faster for those with higher baseline MMR). MMR average and inequality gaps –absolute (AIG), and relative (RIG)– were scenario outcomes.

**Results:**

At baseline, MMR was 59.2 per 100,000; AIG was 313.4 per 100,000 and RIG was 19.0 between countries with baseline MMR over twice the global target and those below the regional target. The AARR needed to meet the global and regional targets were -7.60% and -4.54%, respectively; baseline AARR was -1.55%. In the regional MMR target attainment scenario, applying horizontal equity would decrease AIG to 158.7 per 100,000 and RIG will remain invariant; applying vertical equity would decrease AIG to 130.9 per 100,000 and RIG would decrease to 13.5 by 2030.

**Conclusion:**

The dual challenge of reducing maternal mortality and abating its inequalities will demand hefty efforts from countries of the Americas. This remains true to their collective 2030 MMR target while leaving no one behind. These efforts should be mainly directed towards significantly speeding up the tempo of the MMR reduction and applying sensible progressivity, targeting on groups and territories with higher MMR and greater social vulnerabilities, especially in a post-pandemic regional context.

## Background

Maternal mortality is defined as the death of a woman while pregnant or within 42 days of the termination of her pregnancy, irrespective of the duration and site of the pregnancy, due to any cause related to or aggravated by the pregnancy itself or related care, but not due to accidental or incidental causes [1]. Despite sizeable improvements around the turn of the century, maternal mortality continues to pose a grave threat to the reproductive health, wellbeing, and the life of many women globally. Between 1990 and 2015, the time frame set by the Millennium Declaration and its associated Millennium Development Goals, maternal mortality in the Americas sustained a 49% overall reduction (that is, an average change of -2.69% per year) [2], reaching a regional average ratio of 58 maternal deaths per 100,000 live births in 2017 [3].

There are, however, ample and persistent differences in maternal mortality across countries in the Americas, ranging from an average ratio as low as 10 maternal deaths per 100,000 live births in Canada to as high as 480 per 100,000 in Haiti in 2017 [3]. There is evidence that most of these inequalities are ecosocially determined, both between geographic areas and population groups. Across and within countries alike, maternal deaths are disproportionately concentrated in women and territories that are poorer, less educated, or that have less access to sanitation and, more proximally, less access to timely and quality health care services [4–8]. For instance, in 2015 the absolute gap between extreme quintiles of countries according to their human development index was 148 maternal deaths *in excess* per 100,000 live births; as a matter of fact, half of those maternal deaths were disproportionately concentrated in the 20% of countries with less human development, a fact that has not changed since 2000 [4].

The dual challenge of reducing maternal mortality levels and tackling its inequalities has been captured in the Agenda 2030 for Sustainable Development and its Sustainable Development Goals (SDG), which sets a global target of less than 70 maternal deaths per 100,000 live births by 2030, while pledging to *leave no one behind* [9]. Although in the region of the Americas many countries have achieved this global target, there is still an urgent need to address the complex dual challenges of reducing maternal mortality and tackling inequalities. To this end, the Sustainable Health Agenda for the Americas 2018–2030 (SHAA 2030), has set regional targets of less than 30 maternal deaths per 100,000 live births by 2030 [10].

Reaching such a regional target for maternal mortality in the Americas ‒a region of over 1 billion population‒ will demand hefty efforts at the national and local levels especially if, moving beyond averages, a much-needed focus is placed at reducing distributional inequalities in maternal mortality as well. A potentially informative reference of the amount and direction of effort involved in achieving this outcome is given by the *tempo* or pace of change [11] in the maternal mortality ratio over time. Here we developed a set of equity-sensitive scenarios of maternal mortality reduction by 2030 in the Region of the Americas as informed by an array of plausible assumptions about its average annual rate of reduction.

By presenting alternative scenarios on the reduction of the MMR and its distributional inequality by 2030, it will be useful and strategic for policy makers to adjust the actions that are needed to reduce maternal deaths in the countries of the Americas while creating accountability on the commitment to leave no one behind.

## Materials and methods

This analysis is restricted to the 34 countries of the Americas with maternal mortality ratio (MMR) data available from the United Nations’ Maternal Mortality Estimation Interagency Group (MMEIG) [3]. These countries account for over 99.8% of the total number of births in the Americas by mid-2015 [12], and they were the primary units of analysis in this study.

Regional maternal mortality reduction scenarios by the year 2030 were defined based on two core criteria: 1) an intensity factor: the tempo or pace of change in the maternal mortality ratio of each country; and, 2) an equity factor: the distribution of the tempo across countries. The intensity factor equates to the average annual rate of reduction (AARR), which was calculated as [13]:

$$AARR= \frac{ln\left({MMR}_{b}/{MMR}_{t}\right)}{\left({year}_{b}-{year}_{t}\right)} \times 100$$

where *b* means baseline year (i.e., 2015) and *t* means target year (i.e., 2030). Based on this factor, we defined three scenarios: a normative global scenario (scenario A), based on the AARR needed to achieve the global SDG target; a normative regional scenario (scenario B), based on the AARR needed to achieve the regional SHAA target; and a *status quo* scenario (scenario C), empirically based on the actually observed regional AARR in the preceding five years to the baseline.

The equity factor was defined by applying either a proportional or progressive scheme to the AARR distribution across countries. A proportional scheme applies the same AARR to all units of analysis (i.e., a horizontal equity criterion); a progressive scheme applies a greater AARR to countries with higher MMR than to countries with lower MMR (i.e., a vertical equity criterion). Specifically, the progressive scheme applied was defined in two steps: first, from the MMR distribution across countries at the baseline year (i.e., 2015), four strata were identified according to three normatively chosen cutpoints: 140, 70, and 30 maternal deaths per 100,000 live births, respectively. Thus, the four strata were comprised of countries above twice the global target (stratum 1), those below that threshold but above the global target (stratum 2), those below the global target but above the regional target (stratum 3), and those already below the regional target (stratum 4). Second, a unique progressivity pattern was allocated to each strata: a tempo 25% higher for stratum 1; 12.5% higher for stratum 2; 12.5% lower for stratum 3, and 25% lower for stratum 4. The arithmetic mean of these strata-specific AARR satisfies the property of being equal to the overall AARR in each scenario.

Each scenario was designed to generate two outcomes: the impact of the hypothesized changes on the average 2030 MMR (both at the national and regional levels) and on the magnitude of the inequality gap (both absolute and relative) between stratum 1 and stratum 4. The expected 2030 MMR for each scenario was estimated using the following expression [13]:

$${MMR}_{t}= {MMR}_{b}\times {e}^{\left[\left(\frac{AARR}{100}\right) \times \left({year}_{t}-{year}_{b}\right)\right]}$$

where *b* means baseline year (2015) and *t* means target year (2030). The regional absolute inequality gap was defined as the arithmetic difference in the MMR between strata 1 and 4; it represents the number of maternal deaths *in excess* per 100,000 in the stratum of countries with the highest baseline MMR when compared to the stratum of countries with the lowest baseline MMR. The stratum-specific MMR corresponds to the weighted average of the respective country-specific MMRs (weighted by the 2015 live birth population). Analogously, the regional relative inequality gap was defined as the arithmetic quotient between the two stratum-specific MMR values, and it represents the relative risk of maternal death in the stratum of countries with the highest baseline MMR as compared to the stratum of countries with the lowest baseline MMR [13]. These gap inequality measures were used for simplicity on the interpretation, although other type of metrics can be used, such as the slope index of inequality for absolute inequality and the concentration index for relative inequality.

## Results

Table 1 summarizes the six regional maternal mortality reduction scenarios by the year 2030 considered, by combining the two core criteria described –namely, an intensity factor (AARR magnitude) and an equity factor (proportional or progressive AARR cross-country distribution). Normative global scenario A takes account of a global baseline MMR of 219 maternal deaths per 100,000 live births and a global endline MMR of 70 maternal deaths per 100,000 live births (i.e., the global SDG target), rendering an AARR of -7.60%. Normative regional scenario B considers a regional baseline MMR of 59.2 per 100,000 and a regional endline MMR of 30 per 100,000 (i.e., the regional SHAA target), rendering an AARR of -4.54%. Status quo scenario C considers the regional MMR observed between 2010 (64 per 100,000) and 2015 to provide a tempo of -1.55%. Each of these three scenarios has either a proportional or progressive AARR distribution across the four country strata, as shown in said table.

**Table 1:** Six hypothetical maternal mortality reduction scenarios by the year 2030 in the Region of the Americas, as defined by intensity and equity factors and 2015 MMR country strata

2015 MMR strata	classification criteria (cutoff points)	equity factor					
		proportional scheme			progressive scheme		
		normative	normative	status quo	normative	normative	status quo
		global	regional	regional	global	regional	regional
		(scenario A1)	(scenario B1)	(scenario C1)	(scenario A2)	(scenario B2)	(scenario C2)
Intensity factor (AARR, %)		-7.60	-4.54	-1.55	-7.60	-4.54	-1.55
Stratum 1	countries with MMR > 140 × 10^5^	-7.60	-4.54	-1.55	-9.51	-5.67	-1.93
Stratum 2	countries with MMR > 70 ≤ 140 × 10^5^	-7.60	-4.54	-1.55	-8.55	-5.10	-1.74
Stratum 3	countries with MMR > 30 ≤ 70 × 10^5^	-7.60	-4.54	-1.55	-6.65	-3.97	-1.35
Stratum 4	countries with MMR ≤ 30 × 10^5^	-7.60	-4.54	-1.55	-5.70	-3.40	-1.16

Table 2 shows the magnitude of the endline (2030) MMR for each country and scenario studied, as well as the composition of each stratum of countries according to the baseline (2015) MMR. Out of 34, 27 countries have a baseline MMR over the regional 2030 target, including 14 countries above the global 2030 target. As expected, given the intensity of their defining AARRs, all units of analysis have lower endline 2030 MMRs under scenario A than B than C. Likewise, all countries in strata 1 and 2 (i.e., those with higher baseline MMRs) have better outcomes under scenario 2 (progressivity) than under scenario 1 (proportionality).

**Table 2:** Baseline (2015) and endline (2030) maternal mortality ratios by country and MMR reduction scenario. Region of the Americas

Country (*n* = 34)	2015 MMR baseline	2030 MMR					
		scenario A1	scenario B1	scenario C1	scenario A2	scenario B2	scenario C2
Haiti	488	156.0	247.1	387.0	117.3	208.5	365.3
Guyana	172	55.0	87.1	136.4	41.3	73.5	128.7
Bolivia	168	53.7	85.1	133.2	40.4	71.8	125.7
Suriname	122	39.0	61.8	96.8	33.8	56.7	94.0
Saint Lucia	115	36.8	58.2	91.2	31.9	53.5	88.6
Venezuela	115	36.8	58.2	91.2	31.9	53.5	88.6
Guatemala	103	32.9	52.2	81.7	28.5	47.9	79.4
Nicaragua	101	32.3	51.1	80.1	28.0	47.0	77.8
Dominican Republic	94	30.0	47.6	74.6	26.1	43.7	72.4
Peru	94	30.0	47.6	74.6	26.1	43.7	72.4
Paraguay	89	28.4	45.1	70.6	24.7	41.4	68.6
Colombia	85	27.2	43.0	67.4	23.6	39.5	65.5
Jamaica	78	24.9	39.5	61.9	21.6	36.3	60.1
Bahamas	74	23.7	37.5	58.7	20.5	34.4	57.0
Trinidad and Tobago	68	21.7	34.4	53.9	25.1	37.5	55.5
Honduras	67	21.4	33.9	53.1	24.7	36.9	54.7
Saint Vincent and the Grenadines	64	20.5	32.4	50.8	23.6	35.3	52.3
Brazil	63	20.1	31.9	50.0	23.2	34.7	51.4
Ecuador	63	20.1	31.9	50.0	23.2	34.7	51.4
Panama	58	18.5	29.4	46.0	21.4	32.0	47.4
El Salvador	48	15.3	24.3	38.1	17.7	26.5	39.2
Antigua and Barbuda	43	13.7	21.8	34.1	15.9	23.7	35.1
Belize	43	13.7	21.8	34.1	15.9	23.7	35.1
Argentina	41	13.1	20.8	32.5	15.1	22.6	33.5
Cuba	38	12.1	19.2	30.1	14.0	21.0	31.0
Mexico	36	11.5	18.2	28.6	13.3	19.8	29.4
Barbados	31	9.9	15.7	24.6	11.4	17.1	25.3
Costa Rica	28	9.0	14.2	22.2	11.9	16.8	23.5
Grenada	25	8.0	12.7	19.8	10.6	15.0	21.0
Puerto Rico	20	6.4	10.1	15.9	8.5	12.0	16.8
United States of America	18	5.8	9.1	14.3	7.7	10.8	15.1
Uruguay	18	5.8	9.1	14.3	7.7	10.8	15.1
Chile	14	4.5	7.1	11.1	6.0	8.4	11.8
Canada	11	3.5	5.6	8.7	4.7	6.6	9.2

The main outcomes under each scenario explored are presented in Table 3. At the baseline, the regional MMR was (as previously mentioned) 59.2 maternal deaths per 100,000 live births, with a pronounced health gradient across strata. The absolute inequality gap at baseline was 313.4 maternal deaths in excess per 100,000 live births –that is, a MMR 19.0 times higher in strata 1 than strata 4 (i.e., the relative inequality gap). Consistent with the country-level MMR findings, the regional MMR at endline was lower under scenario A than B than C, just as it was the absolute inequality gap. However, reductions in the absolute inequality gap were more pronounced under scenario 2 than 1, regardless of AARR intensity. More saliently, the relative inequality gap in maternal mortality was invariant under scenario 1, whereas under scenario 2 the higher the tempo the higher the inequality attrition.

**Table 3:** Scenario outcomes: aggregate endline (2030) maternal mortality ratios by MMR reduction scenario and corresponding inequality attrition impact. Region of the Americas

Aggregates and inequality gap	2015 MMR baseline	2030 MMR					
		scenario A1	scenario B1	scenario C1	scenario A2	scenario B2	scenario C2
Region	59.2	18.9	30.0	47.0	18.9	29.9	46.8
Stratum 1	330.8	105.7	167.5	262.3	79.5	141.3	247.6
Stratum 2	96.9	31.0	49.1	76.9	26.9	45.1	74.7
Stratum 3	51.0	16.3	25.8	40.5	18.8	28.1	41.7
Stratum 4	17.4	5.6	8.8	13.8	7.4	10.4	14.6
S1-S4 absolute gap	313.4	100.2	158.7	248.5	72.1	130.9	233.0
S1/S4 relative gap	19.0	19.0	19.0	19.0	10.8	13.5	16.9

S1-S4 absolute gap: arithmetic difference in the MMR between Stratum 1 and 4

S1/S4 relative gap: arithmetic quotient in the MMR between Stratum 1 and 4

From a primarily practical standpoint, an alternative, mixed, scenario of MMR reduction with inequality attrition at the regional level by 2030 could be derived. If the 14 countries with baseline MMR over the global SDG target were to speed up to -7.60% their AARR of MMR reduction, and the 13 countries with baseline MMR below the global SDG target but above the SHAA target were to speed up to -4.54% their AARR, while the remaining 7 countries with baseline MMR already below the regional SHAA target were to maintain their AARR of MMR reduction (see Table 2), then the average regional endline MMR would reach 25.9 maternal deaths per 100,000 (meaning the SHAA target would be met), the absolute inequality gap would drop to 91.9 per 100,000 and the relative inequality gap would drop to 7.7. Compared to the other scenarios’ outcomes (as shown in Table 3), this would be the best-case scenario. But reaching this ideal situation will require that countries with baseline MMR above 70 per 100,000 get a tempo almost five times as fast as their 2010–2015 tempo, and those countries with baseline MMR below 70 but above 30 per 100,000 get to double its current tempo.

## Discussion

In this study, we defined a set of equity-sensitive scenarios of maternal mortality reduction by 2030 across countries of the Americas. This was informed by an array of normative assumptions about both the magnitude and distribution of its average speed of change from baseline year 2015.

The finding of greater reductions in MMR associated with faster tempos at country and regional levels is apparent –and conspicuous. Yet the assumption of an average reduction in MMR at a rate of -7.60% per year –implicit in the SDG target to 2030– though desirable, is highly unrealistic in our regional setting. No country in the Americas has attained such speed of change in its MMR in the fifteen years preceding 2015. As a matter of fact, in the five years preceding 2015 –the time frame that informed status quo scenario C– only two countries achieved a AARR faster than -5%: Chile (-7.13%) and Panama (-6.18%), the regional average being at -1.55%. It should be bear in mind that the MMR global target in the 2030 SDG Agenda is aimed, precisely, at those countries with baseline MMR (i.e., 2015) greater than 70 maternal deaths per 100,000 live births: a AARR of -7.60% reflects the amount of effort needed to bring those *at-risk-of-being-left-behind* ratios down to 70 × 100,000 within the allotted 15 years between 2015 and 2030.

If the speed of change observed in the last five years prior to the baseline (i.e., -1.55%) were to continue to 2030, The Americas’ region will seriously miss its MMR own target –regardless of the progressivity of the tempo across countries, as status quo scenarios C1 and C2 show. This region needs a tempo equal to -4.54% to attain the SHAA target of 30 per 100,000 live births in 2030. In other words, the Americas will need –on average– a speed of reduction of its regional baseline MMR three times as fast as the current one to meet its SHAA 2030 target.

Whether the Region of the Americas can speed up simultaneously its pace and progressivity in the reduction of maternal deaths in order to achieve its 2030 targets remains to be seen. One critical aspect in favor of this scenario is that most maternal deaths in the Americas are due to preventable causes: direct obstetrics (e.g. postpartum hemorrhage, pre-eclampsia and hypertensive disorders, pregnancy-related infections, complications of unsafe abortion) deaths represent on average 70% of a regional overall maternal mortality, and indirect obstetrics causes (infectious and non-communicable diseases) represent on average 25% at regional level circa 2019 but this proportion varies considerably across countries^1^.

Our study found consistent proof in favor of applying progressivity to the distribution of the MMR speed of change across countries to improve results in the associated and equally relevant goal of reducing inequalities in maternal mortality at the regional level. All three scenarios with a progressive scheme reduced more markedly the endline MMR absolute inequality gap as compared to those with a proportional scheme and, more importantly, they were the only ones to show reduction of the endline MMR relative inequality gap, the hardest to tackle [14]; alternative scenarios contemplating different degrees of progressivity were considered and similar results were obtained (not presented in this work). To assume that countries may reduce their MMRs at different speeds contingent upon the magnitude of their average MMR (i.e., the higher the average, the faster the speed of change) is not just cogent, but consistent with the principle of fairness [15] and the commitment to leave no one behind of the Agenda 2030 [9]. Moreover, as opposed to proportionality, progressivity takes into account the diminishing returns effect of the relationship between MMR and time: the lower the MMR, the harder to further reduce it (i.e., marginal utility) [14, 16]. This same principle of progressivity can be applied at the subnational levels (states, departments, provinces, regions, etc.) of a country, which would make it possible to reduce inequality within a country; in addition, based on this same principle, numerical targets can be established to reduce inequalities on maternal mortality between subnational levels of a country [13].

Given its exploratory, ecological design, our study has several limitations. A major one relates to the fact that it explored total inequalities as opposed to social inequalities in maternal mortality; in other words, it has not taken into account the social determinants of maternal deaths, save for geography. Yet place –where people live– is a strong predictor of social conditions (and access to health) in the Americas: implicitly, therefore, they may have been accounted for by geography. Another limitation is the low granularity of our study design, using countries as units of analysis to capture the regional setting. But our approach could be –and should be– replicated at lower levels of geographic disaggregation, such as first- and second-order subnational units (i.e., states and provinces). As a third limitation, our study did not explore the intensity of progressivity in the cross-country distribution of the MMR reduction tempo; no recommendation is attached to the fixed –and somewhat arbitrary– progressivity scheme applied, qualitatively useful though to demonstrate the relevance of this criteria to subdue inequalities while improving the average. Another major caveat is that our study did not take into consideration whether the required changes in the speed of MMR reduction in order to attain the global and/or regional targets are commensurate –or not– with the range, cost-effectiveness, and scalability of available public health interventions for prevention and control of maternal mortality; addressing this critical issue will demand further research. Lastly, our study has not account for the short- and long-term expected, allegedly severe, direct and indirect impacts of the COVID-19 pandemic on maternal mortality in the Americas, the most brutally hit region in the world [17, 18].

These constraints notwithstanding, our exploratory scenario analysis seems to leave out of the question the urgency to put in place truly transformative changes if the region is serious about meeting its 2030 targets in maternal mortality by reducing its level and its distributive inequality. Speeding up the rate of reduction while targeting those being left behind must be considered action priorities in a post-pandemic, *building-back-fairer* regional context.

To this end, improving the scope and quality of national and local civil registration and vital statistics, health information, and maternal death surveillance systems; and establishing protocols to search for and analyze each maternal death, and deaths of women of childbearing age suspected of concealing a maternal death, in order to progressively reduce underreporting and misclassification, should be strengthened [19]. Likewise, building institutional capacities for health inequality monitoring should be promoted [20], not just to keep track on the health equity impact of maternal mortality reduction interventions, but to create accountability on the promise to leave no one behind [21]. Countries with the highest levels of maternal mortality continue to have low levels of care by skilled birth attendants and high levels of population with less than 4 prenatal check-ups, indicating that there is still a lack of access to quality care in the region [22].

Ultimately, there is the undeniable need to strengthen primary health care in populations and territories with high degree of social vulnerability [23, 24]. This comprises, for instance, strengthening health workforce competencies and developing tools that allow for the early identification of obstetric risk; identifying and implementing maternal health clinical guidelines based on the best available scientific evidence to enable professionals to improve the quality of care; supporting community strategies that promote quality care for pregnant women with an intercultural approach that brings the population closer to health services; promoting intersectoral work to address sexual and reproductive rights and health with adolescents and youth; and expanding the capacity to care for patients with severe maternal conditions. 

## Data Availability

All data and associated materials can be made available for review upon reasonable request.

## Footnotes

1 PAHO Regional Mortality Database. Update 30 November 2022.
